# Exposure to Secondhand Smoke and Risk of Tuberculosis: Prospective Cohort Study

**DOI:** 10.1371/journal.pone.0077333

**Published:** 2013-10-25

**Authors:** Hsien-Ho Lin, Yi-Ting Chiang, Jen-Hsiang Chuang, Shiang-Lin Yang, Hsing-Yi Chang, Majid Ezzati, Megan Murray

**Affiliations:** 1 Institute of Epidemiology and Preventive Medicine, National Taiwan University, Taipei, Taiwan; 2 Centers for Disease Control, Department of Health, Taipei, Taiwan; 3 Institute of Population Health Science, National Health Research Institute, Miaoli, Taiwan; 4 Department of Epidemiology and Biostatistics, School of Public Health, Imperial College, London, United Kingdom; 5 Department of Epidemiology, Harvard School of Public Health, Boston, United States of America; University of Oxford, United Kingdom

## Abstract

**Background:**

Prospective evidence on the association between secondhand-smoke exposure and tuberculosis is limited.

**Methods:**

We included 23,827 never smokers from two rounds (2001 and 2005) of Taiwan National Health Interview Survey. Information on exposure to secondhand smoke at home as well as other sociodemographic and behavioral factors was collected through in-person interview. The participants were prospectively followed for incidence of tuberculosis through cross-matching the survey database to the national tuberculosis registry of Taiwan.

**Results:**

A total of 85 cases of active tuberculosis were identified after a median follow-up of 7.0 years. The prevalence of exposure to secondhand smoke at home was 41.8% in the study population. In the multivariable Cox proportional hazards analysis, secondhand smoke was not associated with active tuberculosis (adjusted hazard ratio [HR], 1.03; 95% CI, 0.64 to 1.64). In the subgroup analysis, the association between secondhand smoke and tuberculosis decreased with increasing age; the adjusted HR for those <18, > = 18 and <40, > = 40 and <60, and > = 60 years old was 8.48 (0.77 to 93.56), 2.29 (0.75 to 7.01), 1.33 (0.58 to 3.01), and 0.66 (0.35 to 1.23) respectively. Results from extensive sensitivity analyses suggested that potential misclassification of secondhand-smoke exposure would not substantially affect the observed associations.

**Conclusions:**

The results from this prospective cohort study did not support an overall association between secondhand smoke and tuberculosis. However, the finding that adolescents might be particularly susceptible to secondhand smoke's effect warrants further investigation.

## Introduction

Despite the improvement in case detection and treatment, the global incidence of tuberculosis (TB) has not declined substantially over the last decade[1]. In order to reach the goal of global TB elimination by 2050, preventive measures that address determinants of TB are likely to be needed in addition to curative interventions. Tobacco smoke has been identified as an important risk factor for TB because of its high prevalence globally and existing epidemiological literature on its association with active TB[2], [3]. Although active smoking has been consistently shown to increase the risk of TB in numerous epidemiological studies, it remains unclear whether exposure to secondhand smoke is also associated with TB.

Few studies have reported the association between exposure to secondhand smoke and risk of TB. Although most of the studies revealed a positive association, a substantial heterogeneity was found on the observed odds ratios[3]. A previous systematic review compared the studies of secondhand smoke in children and adults, and found that the association between secondhand smoke and TB was particularly strong among children[3]_ENREF_3. However, most of previous studies are retrospective case-control or cross-sectional studies. Using a cohort that is representative of the general population in Taiwan, we investigated the association between secondhand smoke and incidence of active TB. We also estimated the age-specific association between secondhand smoke and TB.

## Methods

### Setting and study subject

Our study population for this investigation was derived from two rounds of large national surveys in Taiwan, National Health Interview Survey (NHIS), conducted in 2001 and 2005 respectively. The NHIS is a periodical, cross-sectional health survey which was carried out jointly by the Bureau of Health Promotion, Department of Health and the National Health Research Institutes in Taiwan[4]. The survey used a multi-stage stratified systematic sampling scheme to select a nationally representative sample of resident population in Taiwan in each round. People living in institutions (e.g., prison and nursing home) and the homeless population were not included in the surveys. The response rate was 94% for the 2001 NHIS and 81% for the 2005 NHIS. The NHIS survey used different questionnaires for those under 12 years old (reported by the care giver) and above 12 years old (self report). Considering the consistency of the content of questionnaire and the scarcity of TB cases in those younger than 12 years of age, we included only those older than 12 years of age in our cohort. Because of the long latency and potential diagnostic delay of TB, we followed the cohort starting from one year after the last survey date in each cohort until development of active TB, death, or December 31^st^ of 2010, whichever came first. Of 33,738 NHIS participants (n = 18,164 in the 2001 wave and 15,574 in the 2005 wave) who were older than 12 years of age and provided personal information, we excluded 8,936 current/former smokers and 30 persons with missing smoking status, 37 people with prevalent TB, 197 persons who died before the start of follow-up. We further excluded 711 persons with missing information on other covariates (see *Measurements of covariates*); a total of 23,827 non-smokers were finally included in the main analysis.

### Measurement of secondhand smoke exposure

In the NHIS questionnaire for those > =  12 years old, exposure to secondhand smoke was self reported by the participants. The 2001 survey contains information on exposure to secondhand smoke at home and the frequency of exposure (number of days exposed per week). In the 2005 survey information was obtained regarding the exposure to secondhand smoke in the previous week before interview and the places (home, workplace, or restaurant, etc) where the exposure occurred. To be consistent in the two surveys, we defined our main exposure as “exposure to secondhand smoke at home” and did not account for exposure in other places.

### Measurement of tuberculosis

The primary outcome of the analysis was incident TB disease. The study participants were followed for incidence of TB through cross-matching the NHIS database to the TB registry of Taiwan Centers for Disease Control (CDC). TB is a notifiable disease in Taiwan; the diagnosis of TB is based on bacteriological evidence or clinical judgment (symptoms/signs, chest X-ray, and response to broad-spectrum antibiotics)[5]. A recent analysis compared the reimbursement database of national health insurance (with 99% coverage) and TB registry during 2005–2007, and found that 96.3% of TB patients in the health insurance database were notified to TB registry[6]. The incidence and mortality of TB in Taiwan has declined substantially in the past decades. More than 50% of the notified cases now come from the elderly population[7].

### Power calculation

We conducted a priori power calculation using the incidence rate of TB in the general Taiwan population (70 per 100,000) and the reported association between secondhand smoke and TB in the previous cohort study (adjusted hazard ratio: 1.49 for active TB and 1.70 for culture-confirmed TB)[7], [8]. The statistical power to detect a significant association between secondhand smoke and TB was estimated to be 69% and 99% if the true relative risk was 1.5 and 2.0 respectively.

### Statistical analysis

We used descriptive statistics to compare baseline characteristics among study participants with and without secondhand smoke exposure. We constructed Cox proportional hazards models to estimate the hazard ratio (HR) for secondhand smoke and active TB, using age as the time scale. We adjusted for potential confounders in the multivariable Cox model based on reported risk factors for active TB in the literature, including sex, age, crowding, household income, marital status, education, alcohol use, and employment status [9], [10], [11]. In order to estimate the HR for tobacco smoke and active TB in those aged <18, > = 18 and <40, > = 40 and <60, and > = 60 respectively, we added three cross-product terms of age and secondhand smoke to the multivariable Cox models. Since a previous systematic review suggested that the association between secondhand smoke and TB might be different in children and adults, we a prior decided to test for multiplicative effect modification by age[3]. We compared the models with and without the interaction terms using the likelihood ratio test. The information on frequency of secondhand-smoke exposure (days per week) was only available for the 2001 survey, and we conducted dose-response analysis in this subgroup. We included only subjects with complete information on all covariates in the main multivariable analysis because the percentage of participants with missing values of any relevant covariates was small (0.2%). All statistical tests were two-sided with an alpha level of 0.05. All confidence intervals were 95%. The data management and the statistical analyses were performed under SAS 9.2 (SAS Institute Inc., Cary, NC).

### Data availability and ethic statement

The NHIS survey data are publicly available through Taiwan National Health Research Institutes. The TB notification data at the individual level are not publicly available. This study was approved by the ethics committee of College of Public Health, National Taiwan University.

## Results

A total of 23,827 non-smokers were followed up for a median duration of 7.0 years (interquartile range: 3.3–7.0), with a total of 125,518 person-years under observation. Eighty-five incident cases of active TB were identified from the TB registry, 58 (68.2%) of which were culture-confirmed cases. The overall incidence rate of active TB in the study population was 68 per 100,000 (95% CI, 55 to 84). The weighted prevalence of exposure to secondhand smoke at home was 61.0% in the 2001 survey and 26.8% in the 2005 survey. At the baseline, participants exposed to secondhand smoke were more likely to be female, uneducated, living in a crowded place, and unemployed compared to unexposed participants (Table 1).

**Table 1:** Distribution of baseline characteristics of the study population by secondhand smoke exposure. Numbers presented are count (percentage) unless otherwise specified.

	Overall (n = 23,827)	Not exposed (n = 13,850)	Second hand smoke (n = 9,977)	Prevalence (%)of second hand-smoke exposure	P Value
Survey					
2001	13,238 (55.6)	6,158 (44.5)	7,080 (71.0)	53.5	<.001
2005	10,589 (44.4)	7,692 (55.5)	2,897 (29.0)	27.4	
Age					
<18	2,908 (12.2)	1,434 (10.4)	1,474 (14.8)	50.7	<.001
> = 18 and <40	10,093 (42.4)	5,794 (41.8)	4,299 (43.1)	42.6	
> = 40 and <60	7,210 (30.3)	4,414 (31.9)	2,796 (28.0)	38.8	
> = 60	3,616 (15.2)	2,208 (15.9)	1,408 (14.1)	38.9	
Sex					
Female	15,084 (63.3)	7,971 (57.6)	7,113 (71.3)	47.2	<.001
Male	8,743 (36.7)	5,879 (42.5)	2,864 (28.7)	32.8	
Education					
College or above	12,158 (51.0)	7,886 (56.9)	4,272 (42.8)	35.1	<.001
High school	9,343 (39.2)	4,814 (34.8)	4,529 (45.4)	48.5	
Less than elementary school	2,326 (9.8)	1,150 (8.3)	1,176 (11.8)	50.6	
Marital status					
Never married	9,139 (38.4)	5,268 (38.0)	3,871 (38.8)	42.4	<.001
Married/co-habitating	12,725 (53.4)	7,345 (53.0)	5,380 (53.9)	42.3	
Divorced/separated/widowed/other	1,963 (8.2)	1,237 (8.9)	726 (7.3)	37.0	
Residing in a crowded home*					
No	21,972 (92.2)	13,218 (95.4)	8,754 (87.7)	39.8	<.001
Yes	1,855 (7.8)	632 (4.6)	1,223 (12.3)	65.9	
Alcohol use**					
Never	19,285 (80.9)	11,049 (79.8)	8,236 (82.6)	42.7	<.001
Social	3,182 (13.4)	2,062 (14.9)	1,120 (11.2)	35.2	
Regular	987 (4.1)	568 (4.1)	419 (4.2)	42.5	
Heavy	373 (1.6)	171 (1.2)	202 (2.0)	54.2	
Employment status					
No	12,003 (50.4)	6,583 (47.5)	5,420 (54.3)	45.2	<.001
Yes	11,824 (49.6)	7,267 (52.5)	4,557 (45.7)	38.5	
Household income					
<NT$30,000 per month	5,113 (21.5)	2,936 (21.2)	2,177 (21.8)	42.4	<.001
NT$30,000–70,000 per month	10,645 (44.7)	5,941 (42.9)	4,704 (47.2)	44.2	
NT$70,000–150,000 per month	6,768 (28.4)	4,134 (29.9)	2,634 (26.4)	38.9	
≧NT$150,000 per month	1,301 (5.5)	839 (6.1)	462 (4.6)	35.5	

* Residing in a crowded home: more than or equal to eight persons per household.

** Never-not current users of alcohol; social-less than once a week; regular-once a week or more and not to the extent of being intoxicated; heavy-once a week or more and to the extent of being intoxicated.

**Figure 1:**
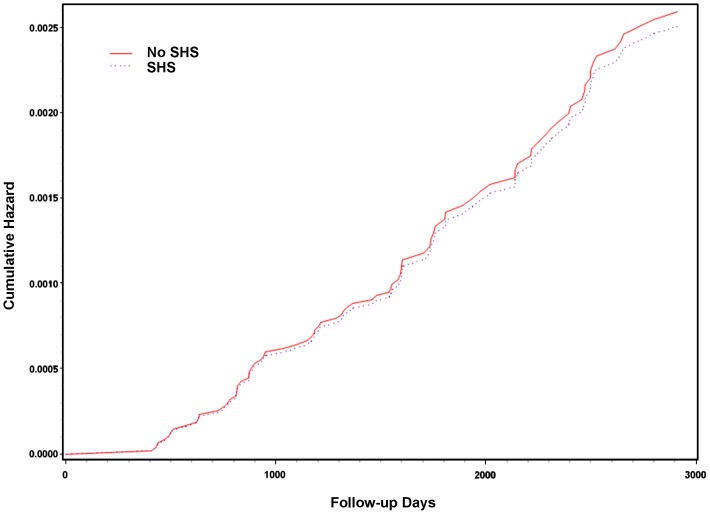
Plot of cumulative hazard of TB among subjects exposed to secondhand smoke (SHS) and among the unexposed subjects.

In the Cox proportional hazards analysis, after adjusting for potential confounders, exposure to secondhand smoke was not associated with active TB (HR = 1.03 (95% C.I. 0.64 to 1.64), Table 2). The plot of cumulative hazard also suggested that the risk for TB was similar among exposed and unexposed groups (Figure 1). The multivariable-adjusted HR was 0.91 (95% CI 0.51 to 1.60) when we restricted the analysis to culture-confirmed cases. In the dose-response analysis using the 2001 survey (65 TB cases among 13,231 participants), there was no significant trend between frequency of secondhand-smoke exposure (days per week) and risk of TB (HR = 0.95 (0.79 to 1.15) for every one day increase in exposure, p = 0.61). In the subgroup analysis, the association for secondhand smoke and TB seemed to be strong in those less than 18 years old (HR = 8.48 (0.77 to 93.56)) and those between 18 and 40 years old (HR = 2.29 (0.75 to 7.01)), and decreased with increasing age (Table 3). However, the result from test of multiplicative effect modification by age did not reach statistical significance (p = 0.059).

**Table 2:** Cox proportional hazards analysis of secondhand smoke exposure, other covariates, and active tuberculosis.

			Age-adjusted				Multivariable*			
	Case number	Pearson-years	HR (95% CI) P Value				HR (95% CI) P Value			
Secondhand-smoke exposure										
No	46	57946	1.00				1.00			
Yes	39	67573	1.14	(0.74,	1.76)	0.54	1.03	(0.64,	1.64)	0.92
Survey year										
2001	65	90443	1.00				1.00			
2005	20	35075	0.82	(0.50,	1.35)	0.44	0.81	(0.48,	1.37)	0.43
Sex										
Female	46	79949	1.00				1.00			
Male	39	45569	1.63	(1.06,	2.50)	0.03	1.62	(0.97,	2.70)	0.07
Education										
College or above	30	63835	1.00				1.00			
High school	29	49823	0.77	(0.48,	1.24)	0.28	0.70	(0.38,	1.28)	0.24
Less than elementary school	26	11861	1.26	(0.75,	2.14)	0.38	1.05	(0.51,	2.16)	0.89
Marital Status										
Never married	9	48465	1.00				1.00			
Married/co-habituating	55	67428	0.90	(0.54,	1.51)	0.70	1.10	(0.33,	3.70)	0.89
Others**	21	9624	1.12	(0.65,	1.95)	0.68	1.51	(0.40,	5.68)	0.56
Residing in a crowded home***										
No	70	115309	1.00				1.00			
Yes	15	10210	2.39	(1.36,	4.19)	0.002	2.52	(1.37,	4.63)	0.003
Alcohol use****										
Never	61	103139	1.00				1.00			
Social	7	15219	0.88	(0.40,	1.93)	0.76	1.02	(0.45,	2.28)	0.97
Regular	10	5136	2.36	(1.21,	4.58)	0.01	2.24	(1.11,	4.52)	0.02
Heavy	7	2025	5.92	(2.70,	13.00)	<.001	5.15	(2.24,	11.84)	<.001
Employment status										
Yes	28	63478	1.00				1.00			
No	57	62040	1.00	(0.57,	1.74)	1.00	0.87	(0.48,	1.55)	0.63
Household income										
≧NT$150,000/month	6	6807	1.00				1.00			
<NT$30,000/month	33	25874	1.42	(1.01,	1.99)	0.05	1.39	(0.50,	3.86)	0.53
NT$30,000 – 70,000/month	28	56362	0.85	(0.61,	1.19)	0.34	0.91	(0.34,	2.47)	0.86
NT$70,000 –150,000/month	18	36476	0.80	(0.54,	1.20	0.28	0.88	(0.32,	2.42)	0.80

* Adjusted for age, survey year, sex, education, marital status, residing in a crowded home, alcohol use, employment status, and household income.

** Others: Divorced/separated/widowed/other.

*** Fewer than eight persons per household, or more than or equal to eight persons per household.

**** Never-not current users of alcohol; social-less than once a week; regular-once a week or more and not to the extent of being intoxicated; heavy-once a week or more and to the extent of being intoxicated.

**Table 3:** Cox proportional hazards analysis of age-specific associations between secondhand smoke and active tuberculosis.

	Age-adjusted		Multivariable*	
	HR (95% CI)	P Value	HR (95% CI)	P Value
<18	7.98 (0.77, 82.97)	0.080	8.48 (0.77, 93.53)	0.081
> = 18 and <40	2.68 (0.88, 8.11)	0.080	2.29 (0.75, 7.02)	0.148
> = 40 and <60	1.36 (0.62, 3.01)	0.440	1.32 (0.58, 3.01)	0.503
> = 60	0.75 (0.41, 1.36)	0.340	0.66 (0.35, 1.23)	0.189

* Adjusted for age, survey year, sex, education, marital status, residing in a crowded home, alcohol use, employment status, and household income.

Other potential risk factors for active TB identified in the multivariable model included male (HR = 1.62 (0.97 to 2.70)), crowding (HR = 2.52 (1.37 to 4.63)), and alcohol use (HR = 5.15 (2.24 to 11.84) for heavy use and 2.24 (1.11 to 4.52) for regular use compared with never user) (Table 2).

## Discussion

To our knowledge, this cohort study is the first to investigate the association between secondhand smoke and incidence of TB in the general population. We did not find a significant association for secondhand smoke and TB in our study population. In the subgroup analysis, however, the adolescents and young adults seemed to be particularly vulnerable to the effect of secondhand smoke on TB. Although more studies are needed to confirm this finding, our results call for further attention to the young population in the ongoing global effort to integrate tobacco control and TB elimination [12].

A limited number of epidemiological studies were conducted on secondhand smoke and the occurrence of TB. One cohort study among female elderly[8], one hospital-based case-control study[13], and one case-control study in close contacts of smear-positive TB patients[14] reported a positive association between secondhand smoke and active TB in adult population. The reported relative risk ranged from 1.49 (1.01 to 2.19) in the cohort to 2.37 (0.94 to 6.01) and 2.5 (1.0 to 6.2) in the case-control studies. Two case-control studies have been conducted specifically in children, and the estimated odds ratio for secondhand smoke and active TB were considerably higher (5.39 (2.44 to 11.91) and 9.31 (3.14 to 27.58) respectively)[15], [16]. Since most of the previous studies were case-control studies, the results can be biased due to differential recall of exposure status among cases and controls and inadequate selection of control population.

Unlike previous studies of secondhand smoke and TB, we did not find an overall association between secondhand smoke exposure and risk of TB[3], [8]. Recent data from the Adult Smoking Behavior Surveillance System suggested that the prevalence of current smoking and secondhand smoke exposure has declined during the past decade in Taiwan [17]. Since the measurement of secondhand-smoke exposure was only made once at the start of follow-up and was based on self-report, the exposure misclassification could have resulted in underestimation of the effect of secondhand smoke. Our sensitivity analysis, however, suggests that this exposure misclassification would have at most resulted in small to moderate underestimation of the true effect of secondhand smoke (see Text S1). In the addition, we conducted a separate analysis on the association between secondhand smoke and a health outcome that is known to be affected by secondhand smoke, ischemic heart disease. We found that the magnitude of association between secondhand smoke and ischemic heart disease in our study population (multivariate-adjusted HR: 1·35 (0.91 to 2.01)) was similar to that reported in a recent meta-analysis (relative risk  = 1·27 (1.19 to 1.36)) (Text S1)[18].

Our subgroup analysis revealed a possible age gradient for the association between secondhand smoke and active TB, with the adolescents being the highest risk group. Given the small number of TB cases in each age group, the confidence intervals of age-specific associations were all wide and the result from statistical test of effect modification by age was not significant. We therefore conducted a separate analysis using all participants of NHIS to estimate the age-specific associations between active smoking and TB; we found that the association was also strongest in adolescents (HR  = 22.94 (4.14 to 127.09)) (Text S2 and Table S2). The exact biological mechanism for the age-dependent association between tobacco smoke and TB remains to be elucidated. The immune response related to host protection against TB, such as the macrophage function and the IL-12/IFN-gamma circuit, is matured in adolescence and therefore should be similar in all age groups of our study population[19]. One hypothesis for the differential susceptibility is that tobacco smoke might be associated with increased risk of primary progression rather than reactivation from remote infection. The prevalence of TB in Taiwan has declined rapidly in the past decades[20]. Since active TB in adolescents is more likely due to primary progression from recent infection, the increased relative risk in adolescents suggests that the hazard of tobacco smoke is mediated through primary progression rather than remote reactivation. Further studies are needed to confirm this age differential association between tobacco smoke and TB in other populations and to understand the underlying biological mechanism.

A major strength of our study is the use of large health surveys to assemble a cohort and cross linkage to national TB registry with complete coverage nationwide. As the participation rate was high in both waves of NHIS (94% in 2001 and 81% in 2005), the possibility of selection bias was small. Since the collection of exposure information was done before the occurrence of disease, the chance of differential misclassification of secondhand-smoke exposure is small. In addition, the detailed personal information collected at baseline enabled us to adjust for major risk factors for TB in the analysis.

One limitation of the study is the small number of TB cases in each age group, particularly in the younger age groups because of the low incidence of TB. Therefore the wide confidence intervals in the age-specific analysis were consistent with both harmful and protective effect of secondhand smoke. However, the strong association in adolescents was found in separate analyses of active smoking and secondhand smoke, indicating that random variation (chance) alone could not explain the results. In Taiwan, a comprehensive smoke-free policy in all enclosed workplaces and public places was mandated in July 2007. Our participants might have been exposed to secondhand smoke in the workplaces and public places prior to 2007. We noted that the exposure of secondhand smoke outside household could have biased the effect estimate toward the null, but we do not think that this could explain the observed decrease in HR with increasing age. If there was exposure to secondhand smoke in the workplaces, we would expect to see a stronger association for secondhand smoke and TB in the youngest (<18) and the oldest (> = 60) age groups, and a weaker association in the middle (> = 18 and <40, > = 40 and <60) age groups.

The results from our study added to the limited evidence on the association between secondhand smoke and TB, and further indicated that the effect of smoking might not be homogeneous across all age groups. Importantly, exposure to tobacco smoke might substantially increase the risk of TB in adolescents. Recently WHO and the International Union Against Tuberculosis and Lung Disease called for a coordinated effort to integrate TB and tobacco control[21]. Further studies are needed to determine whether the young population should be given priority in this ongoing global effort.

## Supporting Information

Figure S1 Bias analysis on the potential impact of declining exposure over time on the observed association between secondhand-smoke (SHS) exposure and active TB
(TIF)

Table S1 Cox proportional hazards analysis of secondhand smoke exposure and coronary heart disease
(DOCX)

Table S2 Cox proportional hazards analysis of age-specific associations between active smoking and tuberculosis
(DOCX)

Text S1 Sensitivity analysis on the bias due to misclassification of secondhand smoke exposure
(DOCX)

Text S2 Analysis of the age-specific association of active smoking and tuberculosis
(DOCX)
