# Supplementary figures and images for: Exposure to Secondhand Smoke and Risk of Tuberculosis: Prospective Cohort Study
Source: PLoS One. 2013 Oct 25;8(10):e77333. doi: 10.1371/journal.pone.0077333 (PMC3808396; doi:10.1371/journal.pone.0077333)

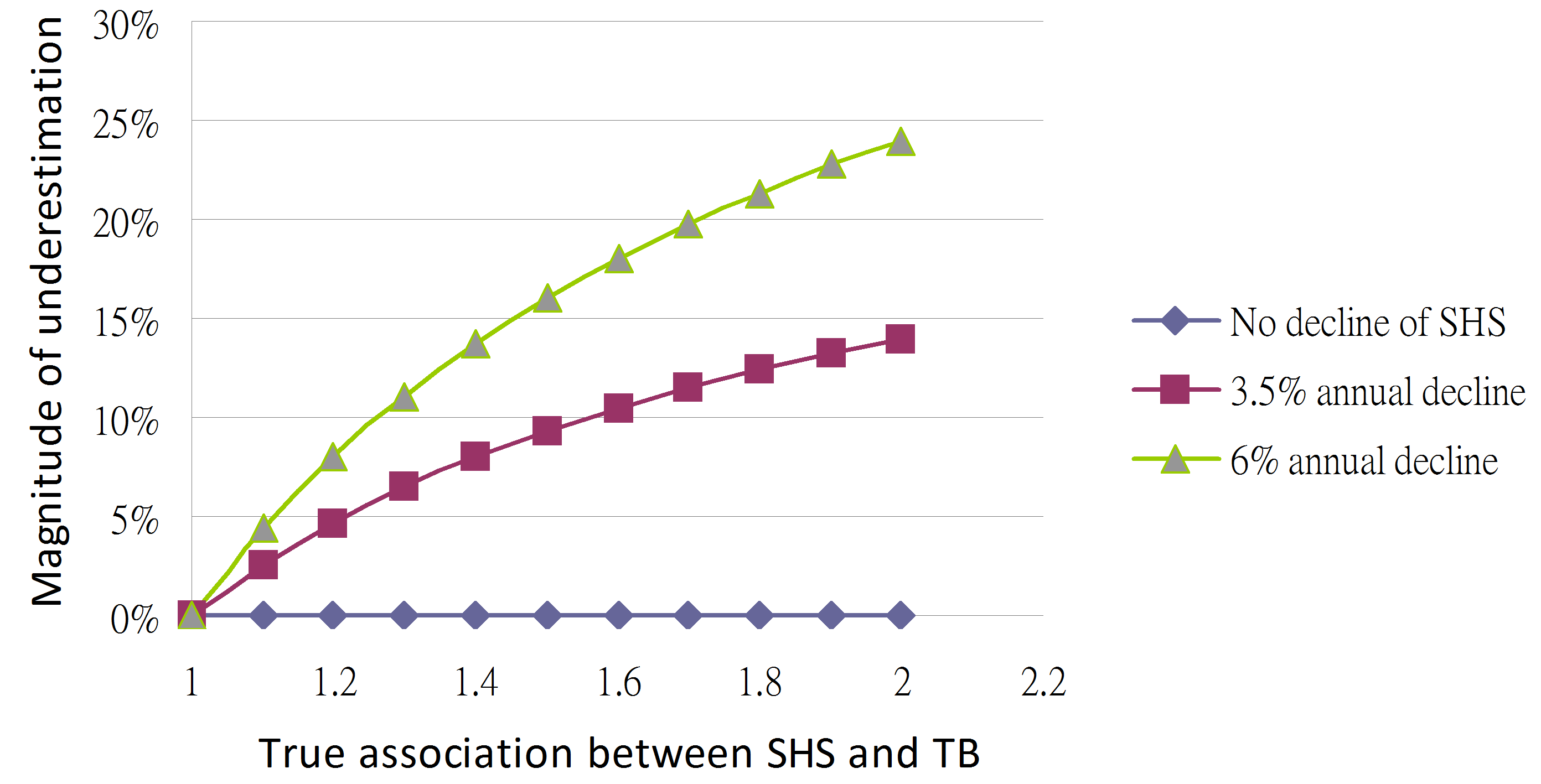

Supplement: Figure S1 — Bias analysis on the potential impact of declining exposure over time on the observed association between secondhand-smoke (SHS) exposure and active TB (TIF) [file pone.0077333.s001.tif]
