# Supplementary material for: Exposure to Secondhand Smoke and Risk of Tuberculosis: Prospective Cohort Study
Source: PLoS One. 2013 Oct 25;8(10):e77333. doi: 10.1371/journal.pone.0077333 (PMC3808396; doi:10.1371/journal.pone.0077333)
Supplement: Table S1 — Cox proportional hazards analysis of secondhand smoke exposure and coronary heart disease (DOCX) [file pone.0077333.s002.docx]

**Table S1.** Cox proportional hazards analysis of secondhand smoke exposure and coronary heart disease

|  | **Univariable^*^** | | | | | | |  | **Multivariable^**^** | | | | | | |
| --- | --- | --- | --- | --- | --- | --- | --- | --- | --- | --- | --- | --- | --- | --- | --- |
|  | **HR (95 % CI)** | | | | | | **P Value** |  | **HR (95 % CI)** | | | | | | **P Value** |
| Secondhand smoke | 1.40 | ( | 0.96 | , | 2.02 | ) | 0.08 |  | 1.35 | ( | 0.91 | , | 2.01 | ) | 0.14 |
| Survey year |  |  |  |  |  |  |  |  |  |  |  |  |  |  |  |
| 2001 | 1.00 |  |  |  |  |  |  |  | 1.00 |  |  |  |  |  |  |
| 2005 | 0.92 | ( | 0.62 | , | 1.36 | ) | 0.67 |  | 0.94 | ( | 0.62 | , | 1.41 | ) | 0.75 |
| BMI |  |  |  |  |  |  |  |  |  |  |  |  |  |  |  |
| <18.5 | 1.60 | ( | 0.77 | , | 3.31 | ) | 0.21 |  | 1.84 | ( | 0.85 | , | 3.99 | ) | 0.12 |
| >=18.5 and <23.0 | 1.00 |  |  |  |  |  |  |  | 1.00 |  |  |  |  |  |  |
| >=23.0 and <25.0 | 0.68 | ( | 0.42 | , | 1.09 | ) | 0.11 |  | 0.85 | ( | 0.50 | , | 1.46 | ) | 0.56 |
| >=25.0 and <30.0 | 1.36 | ( | 0.92 | , | 2.01 | ) | 0.12 |  | 1.39 | ( | 0.88 | , | 2.20 | ) | 0.16 |
| >=30 | 1.90 | ( | 0.96 | , | 3.77 | ) | 0.07 |  | 1.95 | ( | 0.93 | , | 4.10 | ) | 0.08 |
| Male | 1.03 | ( | 0.70 | , | 1.50 | ) | 0.89 |  | 1.21 | ( | 0.78 | , | 1.87 | ) | 0.40 |
| Education |  |  |  |  |  |  |  |  |  |  |  |  |  |  |  |
| College or above | 1.00 |  |  |  |  |  |  |  | 1.00 |  |  |  |  |  |  |
| High school | 0.93 | ( | 0.64 | , | 1.36 | ) | 0.71 |  | 1.05 | ( | 0.62 | , | 1.76 | ) | 0.86 |
| Less than elementary school | 1.28 | ( | 0.84 | , | 1.96 | ) | 0.25 |  | 1.29 | ( | 0.70 | , | 2.36 | ) | 0.42 |
| Marriage Status |  |  |  |  |  |  |  |  |  |  |  |  |  |  |  |
| Never married | 1.00 |  |  |  |  |  |  |  | 1.00 |  |  |  |  |  |  |
| Married/co-habitating | 1.08 | ( | 0.69 | , | 1.69 | ) | 0.73 |  | 1.24 | ( | 0.31 | , | 4.99 | ) | 0.76 |
| Divorced/separated/widowed/  other | 0.94 | ( | 0.59 | , | 1.50 | ) | 0.80 |  | 1.18 | ( | 0.28 | , | 5.06 | ) | 0.82 |
| Alcohol use |  |  |  |  |  |  |  |  |  |  |  |  |  |  |  |
| Never | 1.00 |  |  |  |  |  |  |  | 1.00 |  |  |  |  |  |  |
| Social | 1.01 | ( | 0.54 | , | 1.90 | ) | 0.97 |  | 0.99 | ( | 0.51 | , | 1.89 | ) | 0.97 |
| Regular | 0.45 | ( | 0.14 | , | 1.42 | ) | 0.17 |  | 0.44 | ( | 0.14 | , | 1.40 | ) | 0.16 |
| Heavy | 2.03 | ( | 0.64 | , | 6.42 | ) | 0.23 |  | 1.73 | ( | 0.53 | , | 5.64 | ) | 0.36 |
| Hyperlipidemia | 1.23 | ( | 0.80 | , | 1.89 | ) | 0.34 |  | 1.22 | ( | 0.79 | , | 1.87 | ) | 0.37 |
| Low-income Household | 1.18 | ( | 0.80 | , | 1.75 | ) | 0.41 |  | 1.21 | ( | 0.81 | , | 1.82 | ) | 0.35 |

* Adjusted for age

** Adjusted for age, survey year, bmi, sex, education, marriage status, alcohol use, hyperlipidemia, and low-income household.
