# Supplementary material for: Exposure to Secondhand Smoke and Risk of Tuberculosis: Prospective Cohort Study
Source: PLoS One. 2013 Oct 25;8(10):e77333. doi: 10.1371/journal.pone.0077333 (PMC3808396; doi:10.1371/journal.pone.0077333)
Supplement: Table S2 — Cox proportional hazards analysis of age-specific associations between active smoking and tuberculosis (DOCX) [file pone.0077333.s003.docx]

**Table S2.** Cox proportional hazards analysis of age-specific associations between active smoking and tuberculosis

|  | **Age-adjusted** | | **Multivariable*** | |
| --- | --- | --- | --- | --- |
|  | **HR (95 % CI)** | **P Value** | **HR (95 % CI)** | **P Value** |
| <18 | 30.29 ( 5.61 , 163.58 ) | <0.001 | 22.94 ( 4.14 , 127.09 ) | <0.001 |
| >=18 and <40 | 2.60 ( 1.27 , 5.30 ) | 0.010 | 1.38 ( 0.65 , 2.96 ) | 0.404 |
| >=40 and <60 | 2.30 ( 1.30 , 4.09 ) | 0.0044 | 1.29 ( 0.70 , 2.41 ) | 0.417 |
| >=60 | 1.79 ( 1.15 , 2.78 ) | 0.0096 | 1.20 ( 0.74 , 1.95 ) | 0.458 |

* Adjusted for age, survey year, sex, education, marriage status, residing in a crowded home, alcohol use, employment status, and household income
