# Supplementary material for: Exposure to Secondhand Smoke and Risk of Tuberculosis: Prospective Cohort Study
Source: PLoS One. 2013 Oct 25;8(10):e77333. doi: 10.1371/journal.pone.0077333 (PMC3808396; doi:10.1371/journal.pone.0077333)
Supplement: Text S1 — Sensitivity analysis on the bias due to misclassification of secondhand smoke exposure (DOCX) [file pone.0077333.s004.docx]

**Text S1. Sensitivity analysis on the bias due to misclassification of secondhand smoke exposure**

Published reports from annual nationwide telephone surveys of adult smoking behavior in Taiwan revealed that exposure to secondhand smoke at household has declined substantially during 2005-2009[1]. Since the measurement of secondhand-smoke exposure was only obtained at baseline of follow-up, the declining trend of exposure would have resulted in underestimation of the observed association between secondhand-smoke exposure and active TB if the effect of former exposure is smaller than that of current exposure. We conducted a bias analysis to quantify the impact of declining exposure over time on the observed association between secondhand smoke and TB. We assumed a baseline prevalence of secondhand-smoke exposure of 50% based on the 2001 NHIS survey result and an incidence rate of 50 per 100,000 per year in the unexposed group based the national notification rate. We explored two scenarios of declining secondhand-smoke exposure, one with annual decline of 3.5% based on the slope of decline in the telephone surveys during 2005-2009, and one with annual decline of 6.0% based on the estimated prevalence of secondhand-smoke exposure in the 2001 and 2005 NHIS. We assumed those with former exposure have no increased risk of TB compared with the unexposed in order to explore the upper bound of the misclassification bias. The result of bias analysis suggests that, under a moderate association between secondhand-smoke and active TB (RR~1.5)[2], the magnitude of underestimation would be 9% if exposure to secondhand smoke had declined by 3.5% annually during the study period and 16% if the annual decline was 6% (Figure S1).

It is also possible that exposure to secondhand smoke was more difficult to quantify through questionnaire. Therefore our results might be subject to exposure misclassification and the association between secondhand smoke and TB might be underestimated. We conducted a separate analysis on the association between secondhand-smoke exposure and a health outcome that is known to be affected by secondhand smoke, ischemic heart disease, in our study population. After adjusting for age, sex, BMI, education, marital status, alcohol use, hyperlipidemia, household income, and survey year, exposure to secondhand smoke was associated a moderate but statistically insignificant risk of coronary heart disease (multivariate-adjusted HR: 1.35 (0.91 to 2.01)) in our study population (Table S1). The magnitude of association was consistent with that reported in a recent meta-analysis (RR=1.27, 95% CI (1.19 to 1.36)) [3]. This lends support to the minimal impact of exposure misclassification on the observed association between secondhand-smoke exposure and TB.
