# Supplementary material for: Exposure to Secondhand Smoke and Risk of Tuberculosis: Prospective Cohort Study
Source: PLoS One. 2013 Oct 25;8(10):e77333. doi: 10.1371/journal.pone.0077333 (PMC3808396; doi:10.1371/journal.pone.0077333)
Supplement: Text S2 — Analysis of the age-specific association of active smoking and tuberculosis (DOCX) [file pone.0077333.s005.docx]

**Text S2. Analysis of the age-specific association of active smoking and tuberculosis**

Our age-specific analysis for secondhand smoke and TB revealed a possible age gradient, with adolescents being the highest risk group. Given the small number of TB cases in each age group, we cannot rule out the possibility of chance in this age-differential pattern. We therefore conducted a separate analysis using all participants of NHIS to estimate the age-specific associations between active smoking and TB.

The original cohort consisted of 33,738 NHIS participants (n=18,164 in the 2001 wave and 15,574 in the 2005 wave) who were older than 12 years of age. After excluding those with missing smoking information, we further excluded subjects who had prevalent TB, who died before the start of follow-up, and whose information on other covariates was missing. A total of 32,408 subjects were included in the analysis of active smoking and TB. Similar to the analysis of secondhand smoke and TB, we found that adolescents were particularly at risk for the effect of tobacco smoking: the HR for active smoking and active TB among those <18, >=18 and <40, >=40 and <60, and >=60 years old was 22.94 [4.14 to 127.09], 1.38 [0.65 to 2.96], 1.29 [0.70 to 2.41], and 1.20 [0.74 to 1.95], respectively (p value for effect modification by age: 0.07) (Table S2).
